# Multidimensional Assessment of Health-Related Quality of Life in Ethiopian Children with Scoliosis: Evidence from WHODAS 2.0 and EQ-5D-Y

**DOI:** 10.3390/jcm15166311

**Published:** 2026-08-15

**Authors:** Reta Wakoya, Mekbeb Afework, Alemayehu Worku, Firaol Dandena, Stefano Bolongaro, Yohannis Nigusu, Naol Jigi, Timothy Nunn

**Affiliations:** 1 Department of Biomedical Science, Menelik II Medical and Health Science College, Addis Ababa 1000, Ethiopia; 2 Department of Anatomy, School of Biomedical and Laboratory Medicine, College of Health Science, Addis Ababa University, Addis Ababa 1000, Ethiopia; m.afework@yahoo.com; 3 School of Public Health, Addis Ababa University, Addis Ababa 1000, Ethiopia; alemayehuwy@yahoo.com; 4 CURE Children’s Hospital of Ethiopia, Addis Ababa 1000, Ethiopia; firaol.dandena@cureinternational.org (F.D.); stefano.bolongaro@cureinternational.org (S.B.); yohannis.nigusu@cureinternational.org (Y.N.); mamejigy@gmail.com (N.J.); tim.nunn@cureinternational.org (T.N.)

**Keywords:** scoliosis, health-related quality of life, WHODAS 2.0, EQ-5D-Y, pediatric epidemiology, Ethiopia

## Abstract

**Background/Objectives:** Scoliosis in children leads to complex physical, psychosocial, and functional impairments, yet evidence from low-resource settings is limited. To evaluate health-related quality of life (HRQoL) in Ethiopian children with scoliosis using WHODAS 2.0 and EQ-5D-Y, and to identify clinical and demographic correlates of disability. **Methods:** A hospital-based cross-sectional study was conducted at CURE Children’s Hospital of Ethiopia (1 April 2024–31 March 2025). Ninety-seven children aged 6–15 years with confirmed scoliosis were assessed. WHODAS 2.0 (36-item) captured disability across six domains, while EQ-5D-Y measured physical, psychosocial, and pain-related HRQoL. Clinical severity indicators (Cobb angle, trunk rotation, thoracic morphometrics, anthropometrics) were recorded. Analyses in Python 3.12 included descriptive statistics, correlation, and multiple regression to examine associations between scoliosis severity and HRQoL outcomes. **Results:** Ninety-seven Ethiopian children diagnosed with scoliosis (mean age 11.4 years, mean Cobb angle 73.5°) were assessed; congenital scoliosis was most common (45.4%), followed by idiopathic (38.1%) and neuromuscular (16.5%). WHODAS 2.0 revealed substantial disability, particularly in mobility (46.4%) and social participation (62.8%), with life activities and participation as the strongest contributors. Greater deformity, younger age, and smaller body size were linked to worse outcomes, with neuromuscular and very severe thoracolumbar cases most affected. EQ-5D-Y showed marked HRQoL impairments across domains, with psychological distress (mean 2.24/3), pain, and self-care limitations emerging as key burdens. The mean EQ score was 8.89/15 (59.2%), indicating reduced quality of life. Regression analyses highlighted pain, psychological distress, and self-care limitations as the domains most strongly associated with poorer HRQoL, while greater Cobb angle and higher ATR degree were also correlated with reduced quality of life. **Conclusions:** Ethiopian children with scoliosis experience significant multidimensional impairments in HRQoL, with mobility, social participation, pain, and psychological distress as dominant burdens. WHODAS 2.0 and EQ-5D-Y proved complementary in capturing these impacts, supporting their use for early detection, clinical care, and public health planning in resource-limited settings.

## 1. Introduction

### Background

Scoliosis, a three-dimensional spinal deformity, is one of the most common musculoskeletal disorders affecting children and adolescents worldwide, with significant implications for physical and psychosocial health [1]. Beyond its clinical manifestations, scoliosis can impair health-related quality of life (HRQoL), influencing mobility, self-care, social participation, and emotional well-being [2]. In resource-limited settings such as Ethiopia, where awareness, screening, and treatment options remain constrained, understanding the multidimensional impact of scoliosis on children’s lives is critical for shaping effective public health strategies and clinical interventions [3].

Traditional clinical assessments often emphasize radiographic measures and curve severity, yet these fail to capture the broader psychosocial and functional consequences experienced by affected children [4]. To address this gap, validated patient-reported outcome measures have become essential tools in evaluating HRQoL. The World Health Organization Disability Assessment Schedule (WHODAS 2.0) provides a validated, cross-cultural framework for assessing disability across cognition, mobility, self-care, interpersonal relations, life activities, and participation [5]. Initially developed for psychiatric populations, WHODAS 2.0 now encompasses diverse health conditions and has demonstrated adaptability through translations, including recent Amharic validation in Ethiopian trauma cohorts [6,7].

Applying WHODAS 2.0 to scoliosis research in Ethiopia offers a novel opportunity to capture the multidimensional burden of spinal deformity beyond clinical symptoms [7]. Despite extensive global validation, integration with preference-based instruments in pediatric scoliosis remains limited [5]. In Africa, underdiagnosis, cultural perceptions of disability, and disparities in orthopedic care further constrain evidence on psychosocial and functional outcomes [8]. Leveraging WHODAS 2.0 in Ethiopian contexts can generate culturally sensitive data on scoliosis-related disability, strengthening both clinical practice and public health strategies in resource-limited settings. Complementing this, the EQ-5D-Y, a youth-specific adaptation of the EuroQol instrument, offers a standardized measure of HRQoL across five dimensions—mobility, self-care, usual activities, pain/discomfort, and anxiety/depression—and incorporates a visual analogue scale for overall health perception [6,9,10].

No prior research has systematically examined health-related quality of life in Ethiopian children with scoliosis using validated multidimensional tools. Existing studies in Ethiopia focus mainly on clinical severity indices such as Cobb angle or trunk rotation, without linking these measures to psychosocial or functional outcomes. What remains unknown is the full extent of disability and HRQoL impairments in this population, particularly in domains like social participation, pain, and psychological distress. By applying WHODAS 2.0 and EQ-5D-Y in this context, our study provides a nuanced picture of how scoliosis affects children’s daily functioning and well-being. These insights are essential not only for clinical care but also for guiding early screening, rehabilitation strategies, and policy responses to musculoskeletal health challenges in low-resource setting [11]. This study, therefore, aims to evaluate the health-related quality of life of Ethiopian children with scoliosis using WHODAS 2.0 and EQ-5D-Y, providing evidence that bridges clinical outcomes with lived experiences and highlighting areas for targeted intervention.

## 2. Methods

### 2.1. Study Design and Setting

We conducted a hospital-based cross-sectional study at CURE Children’s Hospital of Ethiopia, a tertiary referral center for pediatric orthopedics and spinal deformities, between 1 April 2024, and 31 March 2025. Ninety-seven children aged 6–15 years with scoliosis, clinically diagnosed and radiographically confirmed as part of routine care, were consecutively recruited. The sample size reflected the total number of eligible patients available during the study period; no a priori sample size calculation was performed, as the study was exploratory and population-based within this hospital setting. Health-related quality of life was assessed using two complementary instruments. WHODAS 2.0 (36-item) measured functional disability across six domains: mobility, self-care, cognition, life activities, social participation, and overall functioning, while EQ-5D-Y provided a child-friendly evaluation of mobility, self-care, usual activities, pain/discomfort, and psychological distress. WHODAS items were scored on a 5-point Likert scale and converted to percentage scores, whereas EQ-5D-Y responses were scored on a 3-level scale, with a composite SUM EQ score calculated. This dual-tool approach enabled a multidimensional assessment of scoliosis’s impact, capturing both functional limitations and psychosocial burdens, thereby enhancing clinical relevance and policy applicability in a resource-limited setting.

### 2.2. Study Population and Eligibility Criteria

The study included 97 children aged 6–15 years with scoliosis, clinically diagnosed and radiographically confirmed during routine outpatient visits at CURE Children’s Hospital of Ethiopia between 1 April 2024, and 31 March 2025. Eligibility required enrollment in scoliosis screening, parental consent, and the ability to complete HRQoL assessments (WHODAS 2.0 or EQ-5D-Y), either independently or with caregiver assistance for younger or cognitively limited children. Exclusion criteria were comorbid neurological or cognitive disorders unrelated to scoliosis that could compromise response reliability or refusal to participate. This selection ensured a well-defined pediatric scoliosis cohort while minimizing confounding influences from unrelated health conditions.

### 2.3. Sampling and Study Procedures

Children were screened for scoliosis using the Adam’s Forward Bend Test and scoliometer, with radiographic confirmation based on Cobb angle. Eligible participants were consecutively recruited during routine clinic visits at CURE Children’s Hospital of Ethiopia. Clinical severity was assessed through standardized radiographic measures (Cobb angle, thoracic morphometrics) and physical examination protocols, including angle of trunk rotation. Anthropometric data (age, height, weight, arm span) and socio-demographic variables were collected using structured questionnaires and chart reviews.

Health-related quality of life was evaluated using two validated instruments. WHODAS 2.0 (36-item) measured functional disability across six domains, with scores converted to percentages (0–100%). EQ-5D-Y assessed five domains: mobility, self-care, usual activities, pain/discomfort, and psychological distress, on a three-level scale, with a composite SUM EQ score (range 5–15) expressed as a percentage. Both tools were translated into Amharic, pretested for cultural relevance, and administered by trained assessors through child-friendly interviews, with caregiver assistance when required. Rigorous quality control included double-entry verification and cross-checking outliers against clinical records to ensure data validity.

### 2.4. Radiological Assessment and Measurement Procedure

Cobb angles were measured on standing posteroanterior radiographs by two independent orthopedic surgeons trained in spinal deformity assessment. To ensure reproducibility, both intertester and intra-tester reliability were evaluated. A random subsample of radiographs was remeasured by the same examiner after a two-week interval (intra-tester) and by a second examiner (inter-tester). Intraclass correlation coefficients (ICCs) exceeded 0.90 for both comparisons, indicating excellent reliability of spine measurements.

### 2.5. Statistical Analysis

All analyses were performed in Python 3.12 using Jupyter Notebook 7.0 to ensure reproducibility. Data preprocessing and descriptive statistics were performed using pandas 2.2.2 and numpy 1.26.4, while visualizations were created using matplotlib 3.9.0, seaborn 0.13.2, and plotly 5.22.0. Continuous variables (age, height, weight, arm span, Cobb angle, ATR, thoracic height) were summarized as means with standard deviations, and categorical variables (sex, scoliosis type, curve side, severity) as frequencies and percentages.

For WHODAS 2.0, the dependent variable was the total disability score; independent variables included clinical severity (Cobb angle, ATR, thoracic morphometrics), anthropometry (height, weight, arm span), and categorical factors (sex, scoliosis type, severity). Domain scores were expressed as percentages, with Pearson’s correlations used to assess associations. Multiple regression models (statsmodels 0.14.2) examined domain contributions to overall disability, with fit assessed by R^2^ and F-statistics.

For EQ-5D-Y, the dependent variable was the SUM EQ score; independent variables included the same clinical, anthropometric, and categorical predictors. Domain scores profiled HRQoL impairments, and the composite SUM EQ score was calculated as an unweighted indicator of severity. Regression diagnostics included checks for linearity, residuals, homoscedasticity, multicollinearity (VIF), and influential observations (Cook’s distance).

Pearson’s correlations assessed relationships between scoliosis severity and HRQoL, while multiple regression models identified the strongest predictors of overall impairment. This dual analytic framework enabled parallel evaluation of functional disability (WHODAS 2.0) and psychosocial/pain-related HRQoL (EQ-5D-Y), providing a comprehensive understanding of the scoliosis burden among Ethiopian children.

### 2.6. AI Statement

During the preparation of this manuscript/study, the authors did not use any AI tools or software. The authors take full responsibility for the content of this publication.

## 3. Results

### 3.1. Demographic and Clinical Characteristics

Ninety-seven Ethiopian children with scoliosis (mean age 11.4 years, range 6–15), clinical evaluation, which revealed severe deformity, with a mean Cobb angle of 73.1° (range 19–150°) and a mean trunk rotation of 26.9°. Thoracic height averaged 18.5 cm compared with the expected median T1–T12 height of 21.6 cm, corresponding to a mean thoracic loss of 14.3%. In some cases, thoracic loss approached nearly 49%, underscoring the profound impact of scoliosis on trunk growth and thoracic volume.

The cohort demonstrated a slight female predominance (58.8%). Congenital scoliosis was most common (45.4%), followed by idiopathic (38.1%) and neuromuscular forms (16.5%). The majority of curves were thoracic (86.6%), with right-sided deformities predominating (67.0%). Disease severity was advanced, with 89.7% of patients classified as severe or very severe (Table 1 and Table 2).

### 3.2. WHODAS 2.0 and EQ-5D-Y Outcomes

The WHODAS 2.0 assessment indicated substantial disability, most pronounced in mobility (mean 46.4%) and social participation (mean 62.8%). Cognition, self-care, and life activities showed minimal impairment, while interpersonal functioning was moderately affected. The overall WHODAS score (mean 14.5) reflects substantial associations with limitations in physical mobility and societal participation. The EQ 5D Y outcomes indicated notable impairments in health-related quality of life across all domains. The greatest burden was in psychological distress (“feeling worried, sad, or unhappy,” mean 2.24/3), followed by limitations in usual activities (mean 1.90) and mobility (mean 1.79). The mean SUM EQ score was 8.89/15 (59.2%), presented as a descriptive indicator of relative severity across the five EQ-5D-Y dimensions, but not as a validated percentage of health-related quality of life (Table 3).

WHODAS 2.0 highlighted functional disability and participation restrictions, while EQ-5D-Y emphasized psychosocial distress, pain, and self-care limitations. Together, these instruments revealed that scoliosis imposes a dual burden: restrictions in physical functioning and social engagement, alongside significant emotional and pain-related challenges.

**Table 3:** Comparison of WHODAS 2.0 and EQ-5D-Y outcomes among Ethiopian children with scoliosis (n = 97).

Domain/Dimension	WHODAS 2.0 (Mean % Disability)	EQ-5D-Y (Mean Score, 1–3)	Interpretations
**Cognition**	13.7%	–	Minimal cognitive impairment noted.
**Mobility**	46.4%	1.79	Both tools confirm mobility limitations.
**Self-care**	8.8%	1.34	EQ shows self-care as a major HRQoL predictor.
**Getting along/Interpersonal**	28.1%	–	WHODAS captures social interaction difficulties.
**Life activities/Usual activities**	22.0%	1.90	WHODAS emphasizes daily functioning; EQ confirms activity restrictions.
**Participation (social)**	62.8%	–	WHODAS uniquely captures participation restrictions.
**Pain/Discomfort**	–	1.62	EQ highlights pain as a dominant burden.
**Psychological distress**	–	2.24	EQ uniquely captures the emotional well-being impacts.
**Overall score**	WHODAS total = 14.5	SUM EQ = 8.89/15 (59.2%)	Both confirm and highlight the dual impact of scoliosis, reflecting both physical disability and psychosocial distress.

The WHODAS 2.0 assessment showed that disability related to scoliosis was most pronounced in two areas: mobility (46.4%) and social participation (62.8%). In contrast, cognition and self-care were impacted to a lesser extent. The EQ-5D-Y assessment highlighted significant impairments in health-related quality of life (HRQoL), particularly in psychological distress (2.24/3), usual activities (1.90/3), and mobility (1.79/3), resulting in a mean overall score of 8.89/15 (59.2%). Together, these assessment tools illustrate that scoliosis creates a dual burden; WHODAS emphasizes functional and participation restrictions, while EQ-5D-Y highlights psychosocial distress and limitations related to pain (Table 4).

**Table 4:** WHODAS 2.0 and EQ-5D-Y domain scores and overall outcomes among Ethiopian children with scoliosis (n = 97).

Variable/Domain	N	Mean	SD	Min	25th Percentile	Median	75th Percentile	Max	Missing
**WHODAS 1 Cognition**	97	1.09	1.70	0	0	0	2	7	0
**Relative domain 1 score %**	97	13.66	21.20	0	0	0	25	87.5	0
**WHODAS 2 Mobility**	97	3.71	2.26	0	2	4	6	9	0
**Relative domain 2 score %**	97	46.39	28.30	0	25	50	75	112.5	0
**WHODAS 3 Self-care**	97	0.70	1.35	0	0	0	1	6	0
**Relative domain 3 score %**	97	8.76	16.84	0	0	0	12.5	75	0
**WHODAS 4 Getting along**	97	2.25	2.13	0	0	2	4	8	0
**Relative domain 4 score %**	97	28.09	26.58	0	0	25	50	100	0
**WHODAS 5 Life activities**	97	1.76	1.58	0	0	2	3	6	0
**Relative domain 5 score %**	97	22.04	19.75	0	0	25	37.5	75	0
**WHODAS 6 Participation**	97	5.02	1.67	2	4	5	6	8	0
**Relative domain 6 score %**	97	62.76	20.88	25	50	62.5	75	100	0
**SUM WHODAS (overall disability)**	97	14.54	7.15	3	9	14	18	33	0
**EQ-1 Mobility**	97	1.79	0.48	1	2	2	2	3	0
**EQ-2 Self-care**	97	1.34	0.52	1	1	1	2	3	0
**EQ-3 Usual activities**	97	1.90	0.42	1	2	2	2	3	0
**EQ-4 Pain/Discomfort**	97	1.62	0.55	1	1	2	2	3	0
**EQ-5 Psychological distress**	97	2.24	0.54	1	2	2	3	3	0
**SUM EQ (overall HRQoL score)**	97	8.89	1.37	6	8	9	10	12	0
**Total EQ-5D-Y % (higher = worse)**	97	59.24	9.12	40	53.33	60	66.67	80	0

### 3.3. Comparative Results Narrative

**Figure 1:**
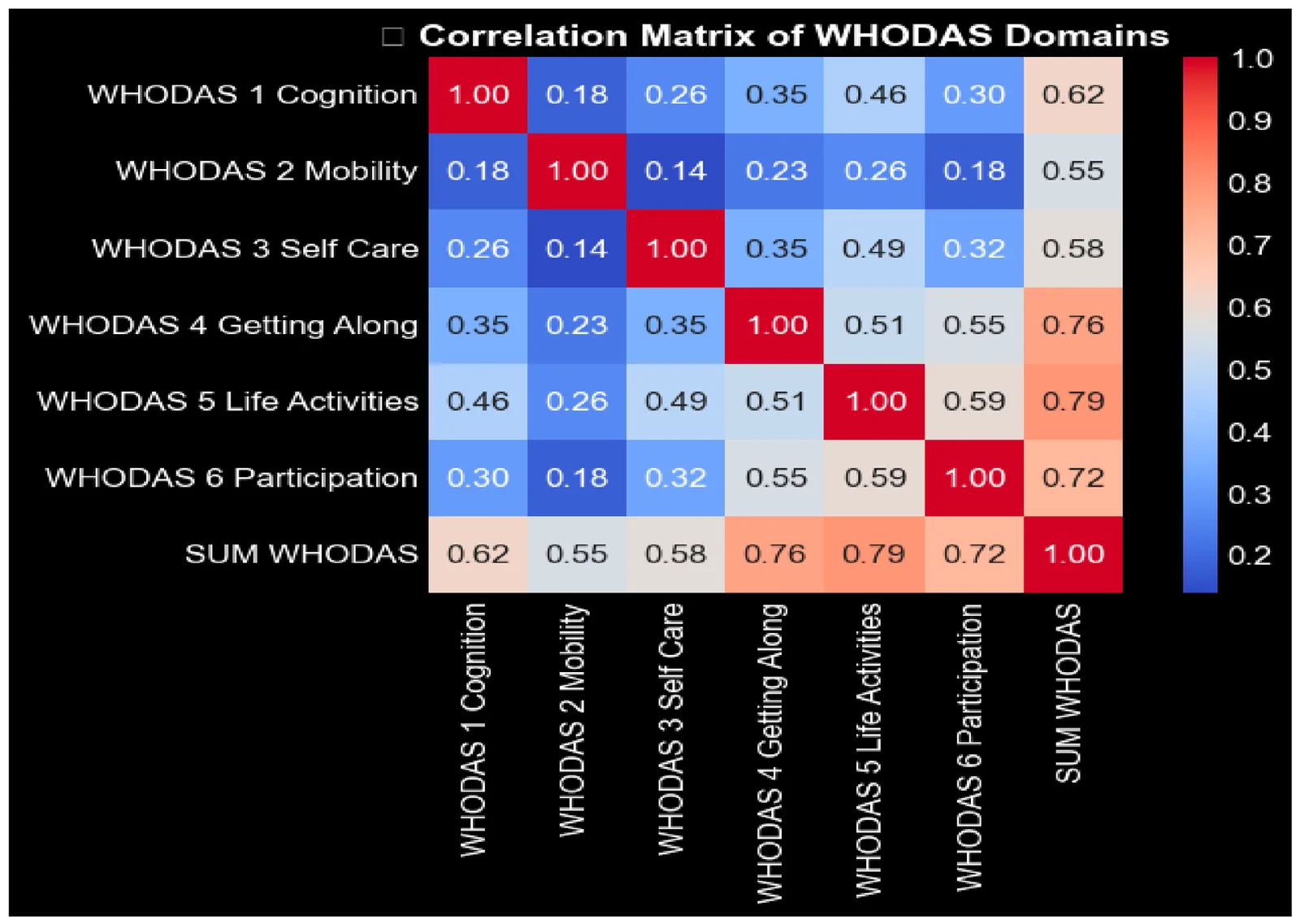
Inter-Domain and Total Score Correlations in WHODAS 2.0 among children with scoliosis.

The WHODAS 2.0 analysis demonstrated that disability in children with scoliosis was most strongly associated with functional and social participation domains, with Life Activities (r = 0.79), Getting Along (r = 0.76), and Participation (r = 0.72) showing the highest correlations with overall disability (Figure 1).

**Figure 2:**
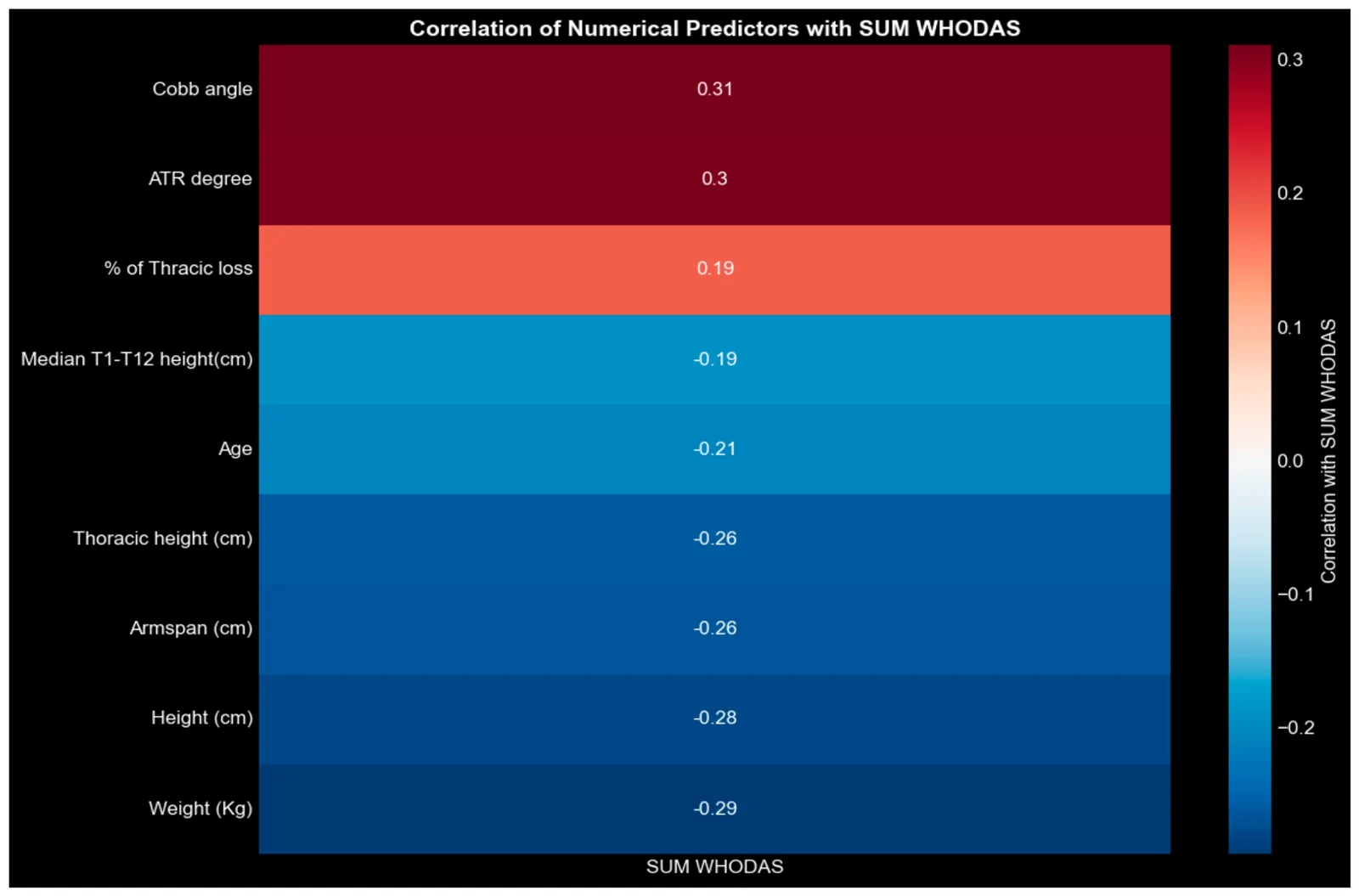
Correlation of clinical and anthropometric predictors with disability scores (SUM WHODAS) in children with scoliosis.

Greater spinal deformity (Cobb angle r = 0.31, trunk rotation r = 0.30, thoracic height loss r = 0.19) was associated with higher disability scores. Smaller anthropometric measures (height, weight, arm span, thoracic height) and younger age showed negative correlations with disability scores, which may reflect age-related differences or underlying disease severity rather than direct growth impairment. Exploratory subgroup analyses indicated that children with neuromuscular scoliosis, thoracolumbar curves, left-sided deformities, and very severe cases had the highest disability scores; however, these findings are descriptive and should be interpreted with caution given small subgroup sizes. Overall, smaller body size and younger age correlated negatively (r = −0.19 to −0.29), suggesting that these characteristics were associated with greater disability, though causality cannot be inferred from cross-sectional data (Figure 2).

**Figure 3:**
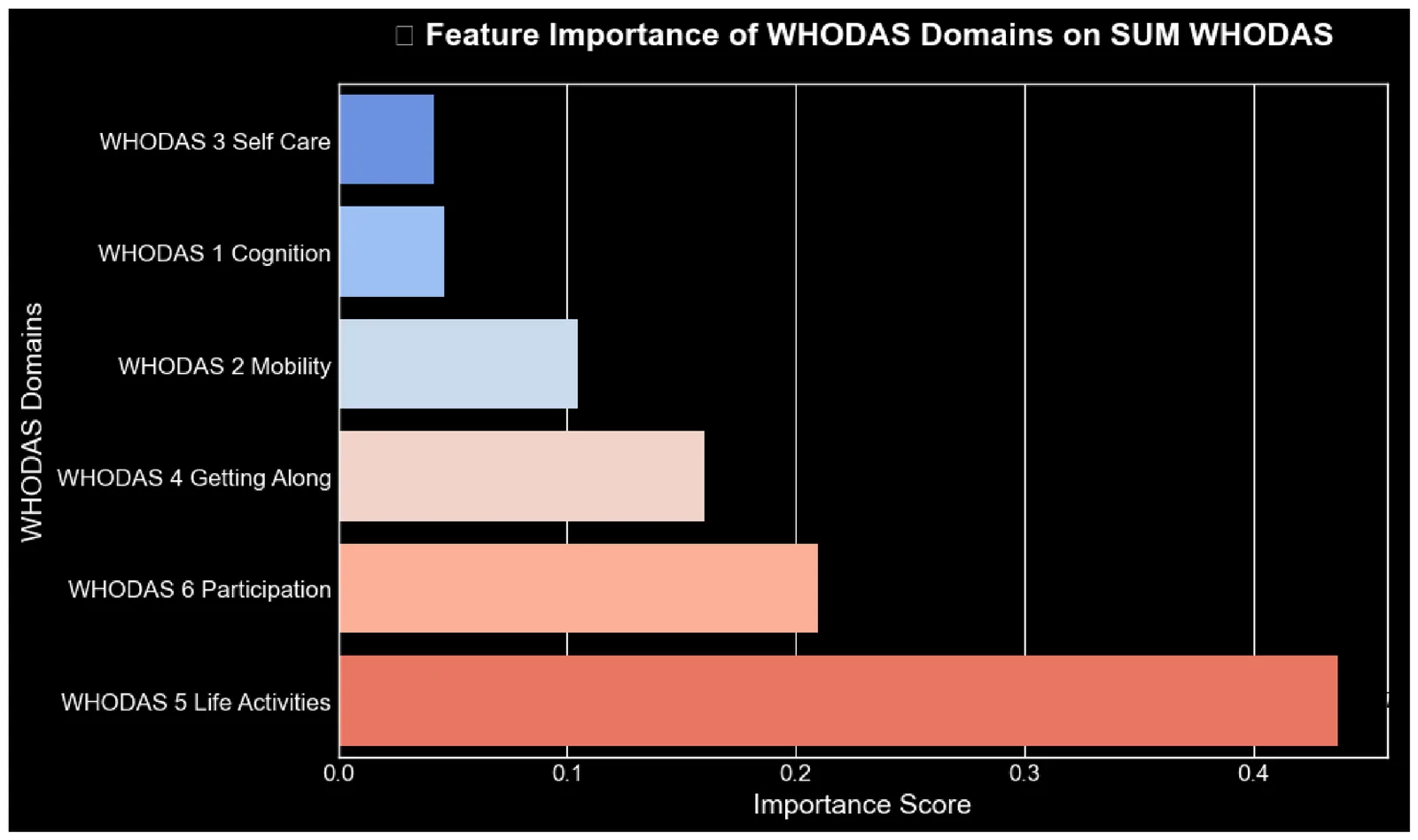
Relative Contribution of WHODAS Domains to Overall Disability Scores in Children with Scoliosis.

A feature importance bar chart analysis confirmed that Life Activities (=0.44) and Participation (=0.21) were the strongest contributors to overall disability, underscoring the central role of daily functioning and social engagement (Figure 3).

**Figure 4:**
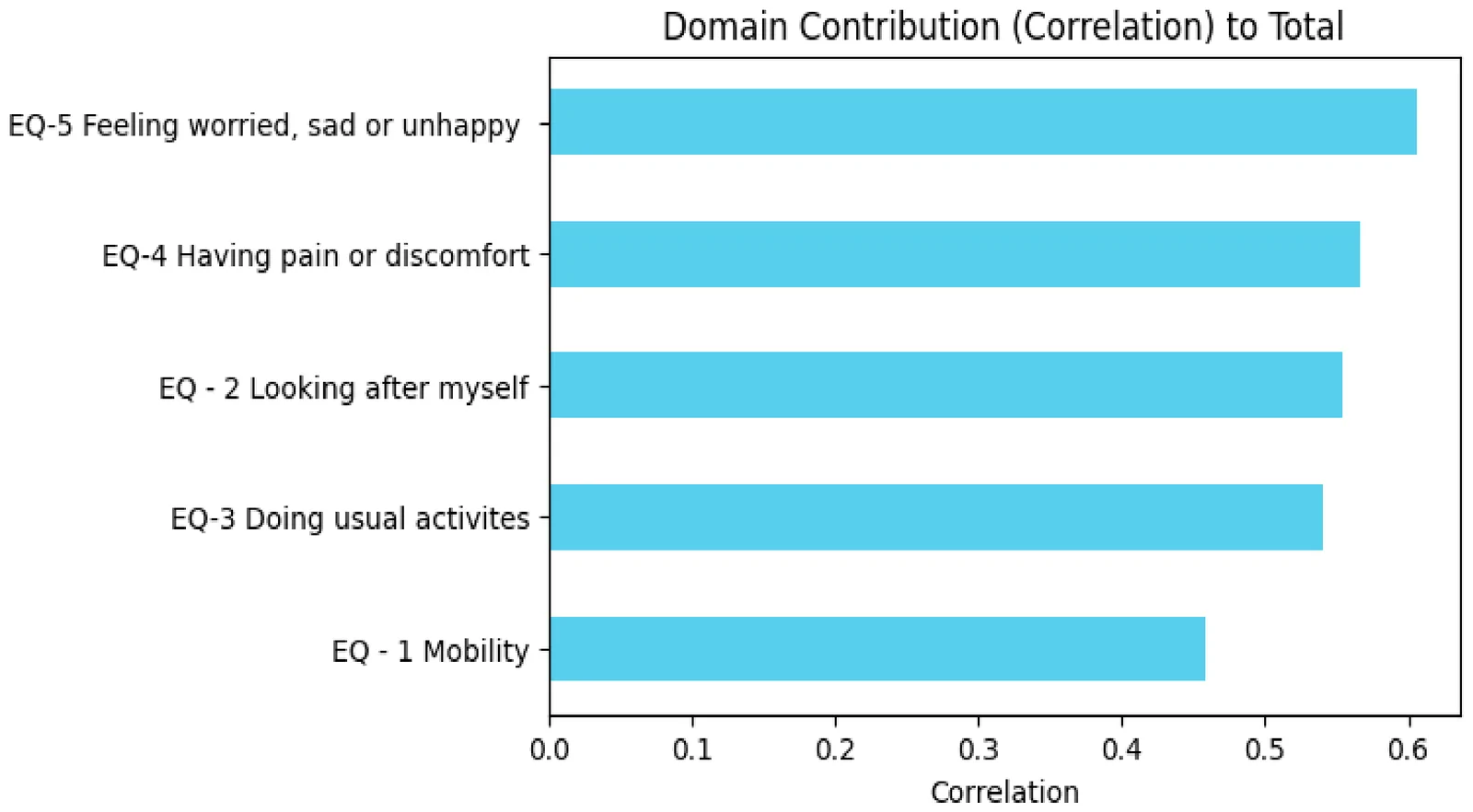
Correlation of EQ-5D-Y Domains with Overall HRQoL Score Among Ethiopian Children with Scoliosis.

In contrast, EQ-5D-Y highlighted psychological distress (mean 2.24/3) and pain/discomfort (mean 1.62) as the most prominent quality of life impairments. Regression analysis confirmed that pain (β = 0.55), psychological distress (β = 0.53), and self-care limitations (β = 0.52) were most strongly associated with overall HRQoL impairment (Figure 4). Overall, the findings from WHODAS 2.0 and EQ-5D-Y indicate that certain domains are independently associated with poorer health-related quality of life (HRQoL) rather than being the strongest predictors.

The perfect R^2^ value of 1.000 is unusually high and may suggest model overfitting or mathematical dependency between the sum score and its components (Table 5). Pain/discomfort (EQ 4, 0.546) and emotional distress (EQ 5, 0.533) emerged as the strongest determinants of overall HRQoL among Ethiopian children with scoliosis. Self-care (EQ 2, 0.516) and mobility (EQ 1, 0.475) showed moderate contributions, while usual activities (EQ 3, 0.418) had the least effect. The intercept (8.887) reflects the baseline HRQoL score. With an R^2^ of 1.000, the model fully explains the variance, highlighting the dominant role of pain and psychological distress, alongside functional limitations, in shaping quality of life outcomes (Table 5).

**Table 5:** Standardized coefficient EQ-5D-Y domain scores and overall outcomes among Ethiopian children with scoliosis (n = 97).

Domain	Standardized Coefficient
**EQ-1 Mobility**	0.4749
**EQ-2 Looking after myself**	0.5155
**EQ-3 Doing usual activities**	0.4183
**EQ-4 Having pain or discomfort**	0.5457
**EQ-5 Feeling worried, sad, or unhappy**	0.5329
**Intercept**	8.8866

**Figure 5:**
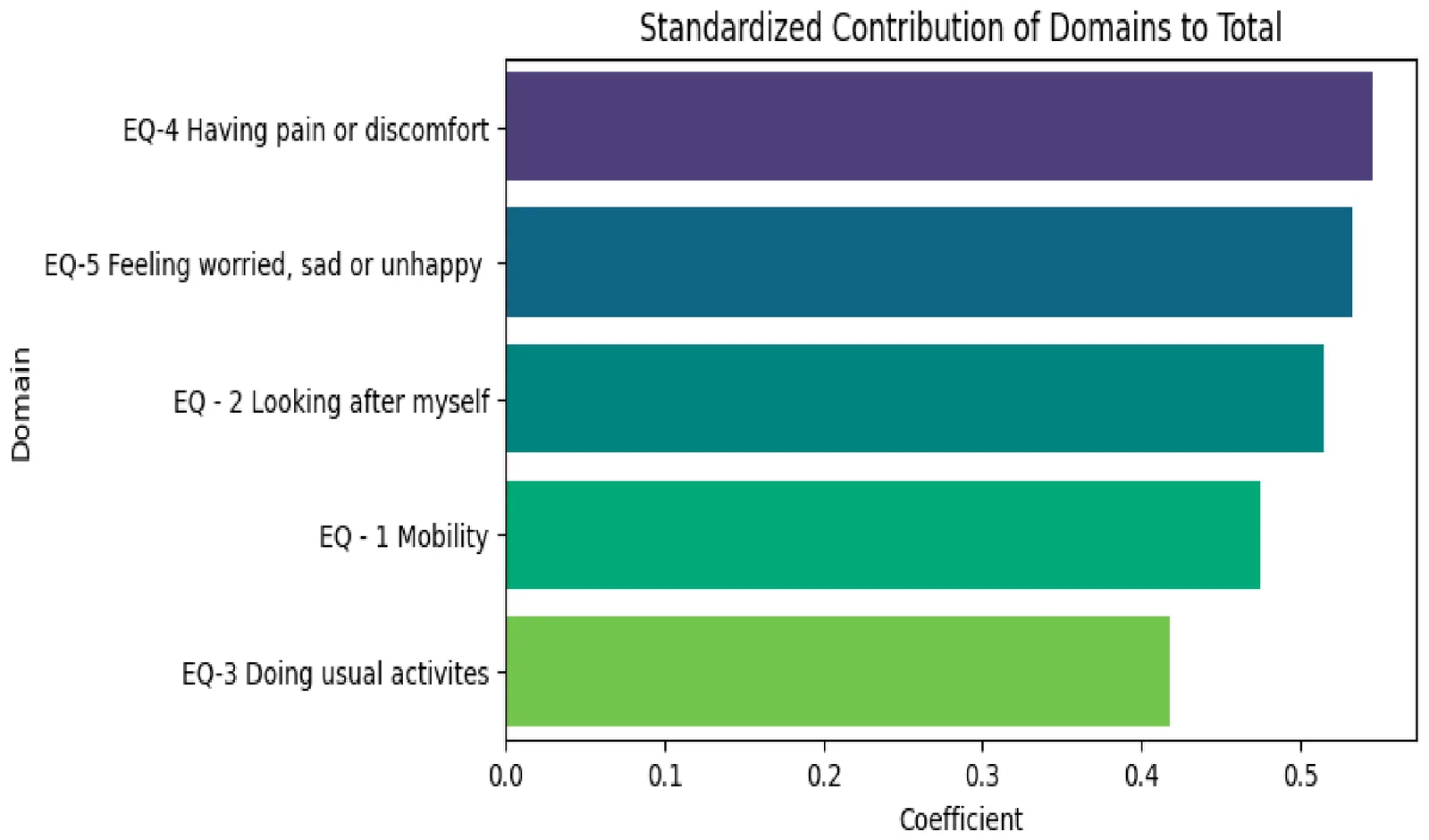
Coefficient of EQ-5D-Y standardized contribution of Domains with Overall HRQoL Score Among Ethiopian Children with Scoliosis.

**Model R^2^ = 1.000** (proportion of variance explained). Pain/discomfort (EQ 4) and emotional distress (EQ 5) were most strongly associated with overall HRQoL impairment among Ethiopian children with scoliosis, reflecting the combined influence of physical and psychological challenges. Mobility (EQ 1) and self-care (EQ 2) showed moderate associations, while usual activities (EQ 3) contributed least. These exploratory findings suggest that interventions addressing pain management and psychosocial support, alongside functional rehabilitation, may be particularly relevant for improving quality of life outcomes (Figure 5).

**Figure 6:**
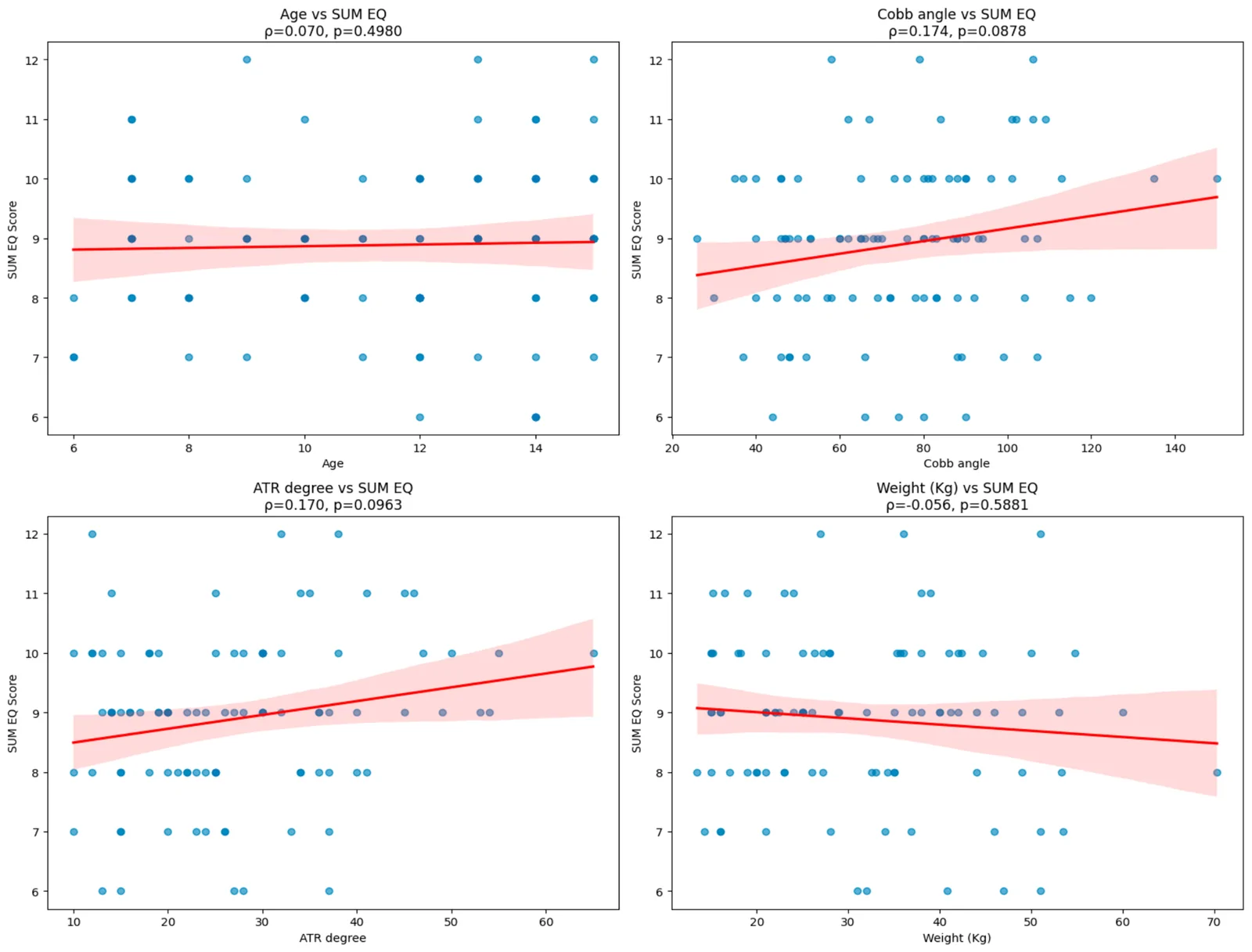
Associations between physical and clinical parameters and HRQoL (SUM EQ Score) in Ethiopian children with scoliosis.

Greater spinal deformity severity (Cobb angle and ATR degree) correlated with worse EQ scores, and subgroup analyses revealed that neuromuscular scoliosis and very severe classifications were associated with the poorest outcomes. The overall SUM EQ score was 8.89/15 (59.2%), reflecting substantially reduced quality of life (Figure 6). Unlike WHODAS, which emphasized functional and participation restrictions, EQ-5D-Y revealed that emotional distress and pain were central to the lived experience of scoliosis.

Together, these findings demonstrate a dual burden: WHODAS emphasizes functional disability and participation restrictions, while EQ-5D-Y underscores psychosocial distress and pain. This complementary perspective highlights the need for integrated interventions that address both physical functioning and emotional well-being in pediatric scoliosis care.

The WHODAS 2.0 assessment revealed that the strongest disability burdens were in participation (62.8%) and mobility (46.4%), with *Life Activities* (=0.44) and *Participation* (=0.21) emerging as the most influential contributors to overall disability. Greater deformity severity, reflected by positive correlations with Cobb angle (r = 0.31), ATR degree (r = 0.30), and thoracic height loss (r = 0.19), was associated with higher disability scores, while smaller body size and younger age correlated negatively, indicating that growth stunting and younger patients reported greater impairment. Subgroup analyses showed that neuromuscular scoliosis, thoracolumbar curves, left-sided deformities, and very severe cases were linked to the highest disability scores. The mean WHODAS score was 14.5, underscoring the dominant burden of scoliosis on physical functioning and social participation.

In contrast, EQ-5D-Y highlighted psychological distress (mean 2.24/3) and pain/discomfort (mean 1.62) as the most prominent quality of life impairments. Regression analysis confirmed that pain (β = 0.5457), psychological distress (β = 0.5329), and self-care limitations (β = 0.5155) were the strongest predictors of overall HRQoL impairment. Greater spinal deformity severity (Cobb angle and ATR degree) correlated with worse EQ scores, and subgroup analyses revealed that neuromuscular scoliosis and very severe classifications were associated with the poorest outcomes. The overall SUM EQ score was 8.89/15 (59.2%), reflecting substantially reduced quality of life (Table 6).

## 4. Discussion

The present study highlights the profound structural burden of scoliosis among Ethiopian children. Yet, clinical indices alone fail to capture the full spectrum of disability, underscoring the need to contextualize these findings through validated patient-reported outcome measures such as WHODAS 2.0 and EQ-5D.

In this cohort of 97 Ethiopian children with scoliosis, clinical evaluation revealed advanced deformity, with a mean Cobb angle exceeding 70° and thoracic loss approaching 49% in severe cases. These findings underscore the profound structural burden of scoliosis in resource-limited settings. However, the functional and psychosocial consequences of such deformity are not fully captured by clinical indices alone, necessitating validated patient-reported outcome measures.

WHODAS 2.0 provides a multidimensional framework for disability assessment, encompassing cognition, mobility, self-care, interpersonal relations, life activities, and participation. In contrast, EQ-5D, as a preference-based measure, evaluates health-related quality of life across mobility, self-care, usual activities, pain/discomfort, and anxiety/depression. While overlapping in scope, WHODAS 2.0 captures broader aspects of social participation and role functioning, which are particularly salient in pediatric scoliosis, where stigma, school engagement, and family dynamics are central. EQ-5D complements this by generating utility-based scores that enable economic evaluation and integration into cost-effectiveness and policy analyses.

International evidence underscores the complementary strengths of WHODAS 2.0 and EQ-5D. WHODAS 2.0 has demonstrated robust cross-cultural validity across diverse health conditions, highlighting its adaptability in low-resource contexts [5,12]. In contrast, EQ 5D has become a cornerstone of health technology assessment, widely applied in musculoskeletal disorders and scoliosis to generate standardized utility scores and enable global comparability [13]. Recent scoliosis studies in Europe and Asia have integrated both tools, showing that WHODAS captures broader disability domains often underestimated by EQ 5D, while EQ 5D provides utility values essential for policy translation [14,15]. Such integration remains rare in African pediatric populations, where underdiagnosis, cultural perceptions of disability, and limited orthopedic services constrain evidence generation.

For instance, the study reported significant HRQoL impairments in European adolescents with idiopathic scoliosis, particularly in mobility and usual activities, findings that parallel those of the Ethiopian cohort [16]. Similarly, validated EQ-5D in Chinese scoliosis patients, underscoring its sensitivity to psychosocial distress and functional limitations [15]. Moreover, the study demonstrated the complementary use of WHODAS and EQ-5D in the Republic of Congo, where WHODAS captured participation restrictions while EQ-5D provided standardized HRQoL measures [17].

In this Ethiopian pediatric scoliosis cohort, WHODAS 2.0 revealed substantial disability, most pronounced in mobility (46.4%) and social participation (62.8%), while cognition, self-care, and life activities showed minimal impairment. These findings underscore that scoliosis disproportionately restricts physical functioning and societal engagement. In contrast, EQ-5D-Y highlighted significant health-related quality of life (HRQoL) impairments, particularly in psychological distress (mean 2.24/3), usual activities (1.90/3), and mobility (1.79/3), resulting in an overall score of 8.89/15 (59.2%). Together, these instruments illustrate a dual burden: WHODAS emphasizes functional disability and participation restrictions, whereas EQ-5D-Y captures psychosocial distress, pain, and limitations in daily activities.

Taken together, these findings highlight the importance of integrating WHODAS 2.0 and EQ-5D-Y in scoliosis research. WHODAS contextualizes the lived experience of disability, particularly in domains of social participation and role functioning, while EQ-5D-Y anchors outcomes within global HRQoL frameworks and facilitates economic evaluation. Their combined use provides a comprehensive assessment of scoliosis burden, bridging functional, psychosocial, and policy-relevant dimensions. This dual approach aligns with international recommendations for multidimensional outcome measurement and strengthens the evidence base for disability-inclusive health policy in Ethiopia and other resource-limited settings.

The comparative analysis of WHODAS 2.0 and EQ-5D-Y in Ethiopian children with scoliosis revealed complementary perspectives on disease burden. WHODAS 2.0 emphasized functional and participation restrictions, with Life Activities (r = 0.79) and Participation (r = 0.72) emerging as the strongest contributors to overall disability, particularly in severe and neuromuscular cases. In contrast, EQ-5D-Y highlighted psychosocial distress and pain as central impairments, with regression analysis confirming pain (β = 0.55), psychological distress (β = 0.53), and self-care limitations (β = 0.52) as key predictors of reduced HRQoL.

These findings parallel international evidence: WHODAS has demonstrated cross-cultural validity and sensitivity to participation restrictions [12], while EQ-5D has consistently captured HRQoL impairments in scoliosis populations across Europe and Asia [13,16]. Studies in China and Sweden reported similar deficits in mobility, usual activities, and psychological domains [15,16]. Recent work in the Republic of Congo confirmed the complementary utility of WHODAS and EQ-5D in capturing both functional and psychosocial burdens [17]. Together, these instruments provide a multidimensional assessment of scoliosis, bridging functional, psychosocial, and policy-relevant perspectives and strengthening disability-inclusive health strategies in resource-limited settings.

Applying both WHODAS 2.0 and EQ-5D in Ethiopian scoliosis research offers a unique opportunity to bridge this gap. WHODAS contextualizes the lived experience of disability, while EQ-5D anchors findings within global HRQoL and economic evaluation frameworks. Together, they provide a comprehensive assessment of disease burden, informing both individualized care and system-level resource allocation. This dual approach aligns with international recommendations for multidimensional outcome measurement and strengthens the evidence base for disability-inclusive health policy in Ethiopia.

### Limitation

This study has several limitations. Its cross-sectional design precludes causal inference and longitudinal assessment, and recruitment from a single tertiary hospital introduces referral bias that limits generalizability. The small, clinically heterogeneous sample and absence of a healthy or mild scoliosis comparison group further constrain interpretation. Proxy response bias in younger children and the lack of validated pediatric Amharic versions of WHODAS 2.0 raise concerns about measurement accuracy. Reliance on an unweighted EQ 5D Y sum score, multiple exploratory analyses, and the modest sample size also increase the risk of model overfitting. These factors underscore the need for cautious interpretation and highlight the importance of future multicenter, longitudinal studies using scoliosis-specific pediatric instruments.

## 5. Conclusions

This study demonstrates that scoliosis in Ethiopian children imposes a dual burden, with WHODAS 2.0 highlighting restrictions in physical functioning and social participation, and EQ-5D-Y emphasizing psychosocial distress, pain, and limitations in daily activities. Greater deformity severity, neuromuscular etiology, and very severe classifications were consistently associated with poorer outcomes across both instruments. These findings parallel international evidence, reinforcing the complementary utility of WHODAS for functional and participation domains and EQ-5D for quantifying HRQoL and policy-relevant utility scores. Integrating both measures provides a multidimensional understanding of scoliosis burden and strengthens disability-inclusive health strategies in resource-limited settings.

## Figures and Tables

**Table 1:** Descriptive characteristics and clinical measurements of Ethiopian children with scoliosis (n = 97) (Anthropometry and deformity measures).

Variable	N	Mean	SD	Min	25%	Median	75%	Max
**Age (years)**	97	11.44	2.81	6	9	12	14	15
**Height (cm)**	97	135.61	19.79	96	118	136	152	176
**Weight (kg)**	97	31.26	12.56	13.5	21	28.1	40	70.3
**Arm span (cm)**	97	141.72	20.89	92	126	142	159	190
**ATR degree**	97	26.94	11.78	10	18	25	34	65
**Cobb angle (°)**	97	73.08	24.66	19	52	73	88	150
**Thoracic height (cm)**	97	18.53	3.93	10	15.4	18.3	21.5	27.8
**Median T1–T12 height (cm)**	97	21.55	2.50	17.2	19.6	21.4	24.3	24.8
**% Thoracic loss**	97	14.26	13.69	−21.93	4.94	13.55	23.83	48.98

**Table 2:** Distribution of scoliosis types and curve severity (n = 97).

Variable	Category	Frequency	Percentage
**Sex**	Female	57	58.8%
	Male	40	41.2%
**Types of scoliosis**	Congenital	44	45.4%
	Idiopathic	37	38.1%
	Neuromuscular	16	16.5%
**Curve type**	Thoracic	84	86.6%
	Thoracolumbar	11	11.3%
	Lumbar	2	2.1%
**Curve side**	Right	65	67.0%
	Left	32	33.0%
**Severity classification**	Severe	54	55.7%
	Very severe	33	34.0%
	Moderate	10	10.3%

**Table 6:** Comparative Summary of WHODAS 2.0 and EQ-5D-Y Analyses.

Aspect	WHODAS 2.0 Findings	EQ-5D-Y Findings
**Strongest domain burden**	Participation (62.8%), Mobility (46.4%)	Psychological distress (2.24/3), Pain (1.62)
**Key contributors**	Life Activities (≈0.44), Participation (≈0.21)	Pain (β = 0.5457), Distress (β = 0.5329), Self-care (β = 0.5155)
**Correlation with severity**	Cobb angle (r = 0.31), ATR (r = 0.30), thoracic loss (r = 0.19)	Cobb angle & ATR are positively correlated with SUM EQ
**Negative predictors**	Smaller body size, younger age	Not emphasized; severity dominates
**Subgroup differences**	Neuromuscular, thoracolumbar, left-sided, very severe	Neuromuscular, very severe classification
**Overall outcome**	Mean WHODAS score = 14.5	SUM EQ = 8.89/15 (59.2%)

## Data Availability

The datasets generated and/or analyzed during the current study are available from the corresponding author upon reasonable request. All data supporting the reported results are included within the published article. No additional publicly archived datasets were created or analyzed. Due to privacy and ethical restrictions related to human participant data, raw individual-level files cannot be made publicly available.

## Abbreviations

ATR	Angle of trunk rotations
EQ	EuroQol Group
EQ-5D-Y	Youth version of the EuroQol five-dimension questionnaire
HRQoL	Health-related quality of life
